# Plant Chemistry and Enemy Pressure Shape Within-Stem Distribution of the Invasive Scale *Nipponaclerda biwakoensis*

**DOI:** 10.3390/insects17010009

**Published:** 2025-12-20

**Authors:** Andrea E. Glassmire, James T. Cronin, Rodrigo Diaz, Alexis DeSoto, Emily Shapiro, Alex Gaffke, Joshua S. Snook, Michael Stout

**Affiliations:** 1 Department of Entomology, Michigan State University, East Lansing, MI 48824, USA; snookjo2@msu.edu; 2 Department of Plant Biology, Michigan State University, East Lansing, MI 48824, USA; 3 Department of Biological Sciences, Louisiana State University, Baton Rouge, LA 70803, USA; jcronin@lsu.edu (J.T.C.);; 4 Department of Entomology, Louisiana State University, Baton Rouge, LA 70803, USA; rdiaz@agcenter.lsu.edu (R.D.); mstout@agcenter.lsu.edu (M.S.); 5 Insect Behavior and Biocontrol Research Unit, Center for Medical, Agricultural, and Veterinary Entomology, Agricultural Research Service, United States Department of Agriculture, Tallahassee, FL 32608, USA; alexander.gaffke@usda.gov

**Keywords:** Aclerdidae, common reed, invasive insect, within-plant dispersal, Growth–Defense Tradeoff, fine-scale dispersal

## Abstract

Invasive insects increasingly threaten ecosystems worldwide, with wetlands especially vulnerable to unpredictable climate. *Phragmites australis* is a dominant plant species in Louisiana’s Mississippi River Delta and a critically important component of the wetland ecosystem. However, the invasive scale insect, *Nipponaclerda biwakoensis*, has contributed to large-scale dieback of this foundation species, jeopardizing erosion control, water filtration, and wildlife habitat. Despite rapid regional spread, the fine-scale dispersal of *N. biwakoensis* within host plants remains poorly understood. We examined whether the crawler-stage of *N. biwakoensis* scales preferentially settled on the bottom or top sections of *P. australis* stems, and whether plant nutritional and/or defensive traits shaped this preference. In field surveys, scale densities varied along the length of *P. australis* stems, with gravid females occurring 3.5× more frequently at the stem base than at the top; parasitism rates were similarly elevated, reaching 12× higher at the base. To evaluate potential drivers of this pattern, we quantified carbon, nitrogen, water, and phenolic content in lower and upper stem tissues and conducted complementary laboratory assays to test crawler settlement preferences. Under controlled conditions, crawlers settled most densely on middle stem sections, with lower densities at the base and the fewest near the top. The basal sections also contained 50% less nitrogen and 47% lower phenolic concentrations compared to the upper stem. The divergence in crawler settlement patterns between field and controlled conditions likely reflects the influence of additional environmental factors present in the field—such as habitat structure, microclimate, and natural enemies—that are absent or minimized in laboratory conditions. By applying a trait-based approach to insect dispersal, we link plant functional traits to *N. biwakoensis* crawler settlement patterns, strengthening our understanding of of insect distribution and guiding predictions of long-term dispersal in *N. biwakoensis*.

## Simple Summary

Wetlands along Louisiana’s Mississippi River Delta depend on a tall grass called *Phragmites australis* to reduce erosion, filter water, and provide wildlife habitat. In recent years, this foundation plant has suffered widespread dieback linked to an invasive insect scale (*Nipponaclerda biwakoensis*). Although this insect spreads rapidly, scientists have known little about how it moves and settles within individual plants—a key step in understanding and managing its impacts. We studied where scale insects settle along the length of *P. australis* stems and why. Field surveys showed that adult scales were far more common near the base of stems, where parasitic wasps that attack the scales were also most abundant. In greenhouse and laboratory experiments, we tested whether plant traits influenced where young scales chose to settle. We found that upper stem sections contained more nitrogen and defensive chemicals, while lower sections were less defended. When given a choice, scale crawlers often preferred the middle and lower sections of the stem. Together, our results show that scale distribution within plants reflects a balance between plant chemistry, habitat structure, and natural enemies. Understanding these fine-scale patterns helps explain how this invasive insect spreads and can inform targeted strategies to slow damage to vulnerable wetlands.

## 1. Introduction

Invasive insects are an increasing global threat, disrupting food webs, outcompeting native species, and altering ecosystem functions across diverse habitats [1,2]. Wetlands are especially vulnerable to insect invasions due to their high biodiversity and frequent human disturbance [3]. In these environments, invasive insects can rapidly establish persistent populations, threatening native species and the vital services wetlands provide, such as water filtration, flood control, and habitat for migratory birds. One key to halting the spread of these harmful invaders is understanding how they disperse at both small and large scales [4,5]. Dispersal patterns reveal where invasive insects are likely to go next, how quickly populations will expand, and which environmental or biological factors facilitate their movement, all of which is essential knowledge for developing targeted, effective management strategies.

Tracking the spread of invasive insects across broad geographic regions offers insights into long-term dispersal and population dynamics, with patterns often revealing multiple introductions, extensive global movement, and widespread population mixing [6]. Equally critical yet often overlooked is understanding insect movement at finer spatial and temporal scales [7,8]. For many herbivorous insects, particularly sap-sucking species like scales, dispersal occurs within the narrow window of mobility that exists during a specific life stage and often takes place entirely within a single plant [9]. In these cases, vertical movement is typically constrained to the plant stem, where individuals decide where to settle and feed. However, herbivorous insects are unevenly distributed within host plants, often reflecting variation among plant parts in quality or microhabitats that provide food resources or shelter from natural enemies [10,11,12]. These patterns raise important questions about the underlying drivers of insect movement—whether it is a random process or shaped by specific plant traits, such as the distribution of nutrients or defenses (chemical or physical), that influence insect settling decisions [13]. Identifying the factors that shape these fine-scale dispersal decisions is crucial for predicting and potentially disrupting the establishment and spread of invasive species

The suitability of a host plant for insects depends primarily on its nutrient profile and defense mechanisms [14,15]. However, these traits are not uniformly distributed within the plant; they often vary among plant organs (e.g., leaves versus stems), and with tissue age and position [13,16,17,18]. Younger, actively growing tissues near the apex of the stem tend to be richer in water and nitrogen—both critical for insect development—whereas older tissues near the base tend to be tougher and lower in nitrogen, traits that can serve to deter herbivorous insects [19,20,21]. Defensive traits, such as phenolic compounds that deter feeding and reduce digestibility in insects [22,23] can also vary from the bottom to the top of the same stem. Phenolic concentrations are often elevated in younger leaves or leaves exposed to high light or UV radiation, suggesting a localized protective function [16,24,25]. Such within-plant variation, particularly along the length of the stem, may strongly influence fine-scale dispersal and settling decisions by insects. Quantifying these gradients in plant quality, and how they relate to insect settlement decisions, is essential for understanding how insects navigate and utilize their immediate environment.

*Nipponaclerda biwakoensis* (Kuwana) (Hemiptera: Aclerdidae) provides an ideal model for investigating the factors influencing fine-scale dispersal of a scale insect across the stem length of its host plant, *Phragmites australis* (Cav.) Trin. ex. Steudel. In Louisiana’s Mississippi River Delta, *P. australis* is a dominant wetland grass that plays a critical role in reducing erosion, buffering storm impacts, and supporting wildlife [26,27,28]. However, the decline of *P. australis*, first detected in 2016, is concerning, as reduced coverage weakens its role in controlling soil erosion and preventing land loss [28,29,30]. Notably, the invasive scale insect *N. biwakoensis*, native to East Asia, was first detected in the Mississippi River Delta (MRD) in 2016 and has been identified as a major contributor to *P. australis* dieback [28,31,32]. Current evidence indicates that top-down control via parasitoids may be one of the most important mortality factors of this invasive scale [33]. Since its initial detection, *N. biwakoensis* has rapidly spread across coastal Louisiana and into neighboring states including Texas, Mississippi, and Alabama [28,34]. Despite its alarming expansion, key gaps remain in understanding its dispersal, particularly during its only active dispersal stage, the crawler stage, which lasts approximately 72 h [35]. For many scale insects, including *N. biwakoensis*, the location of crawler settling is important because females will remain immobile for the rest of their life cycle, thus affecting reproduction, the direct interaction with its host plant via feeding, and, indirectly via protection from natural enemies. Also, identifying whether crawlers exhibit settling preferences along the stem length is a critical first step in predicting the subsequent population structure [36]. This apparent vertical population structure could have important implications for interactions with *N. biwakoensis* parasitoids, which are known to partition their niches along the stem [13,28,37].

In this study, we examined the dispersal and distribution of *N. biwakoensis* within stems of *P. australis* to determine whether settling preferences along the length of the stem are potentially influenced by nutritional and defensive plant traits. We first quantified scale insect densities along stems of *P. australis* (Delta haplotype) collected from the Mississippi River Delta. Next, we grew *P. australis* under controlled conditions in a greenhouse to assess variation in carbon and nitrogen content (nutritional traits) and total phenolics (defensive traits) between the bottom and top stem sections. Finally, we conducted two laboratory preference assays to test whether crawler-stage *N. biwakoensis* preferentially settled on the bottom or top stem sections. Our study addressed the following questions: (1) Are field-collected *N. biwakoensis* scale densities greater at the bottom compared to the top of *P. australis* stems? (2) Do nutritional and defensive traits of *P. australis* differ between the bottom and top sections of stems? (3) Do *N. biwakoensis* crawlers prefer settling on the bottom rather than the top stem sections using choice assays? Our results highlight how the fine-scale distribution of *N. biwakoensis* within host stems may shape interactions with its parasitoids. Building on these insights, we recommend strategies to improve predictions of pest spread and to guide targeted control efforts aimed at reducing regional expansion.

## 2. Materials and Methods

### 2.1. Study System

The MRD, where the Mississippi River meets the Gulf of Mexico, is the seventh largest river delta on Earth [38]. Coastal deltas such as the MRD provide immense ecosystem services valued at $12–47 billion annually, including flood protection, water filtration, fisheries, recreation, and cultural and ecological benefits [39]. Yet the MRD has been subjected to a convergence of stressors—coastal erosion, subsidence, hurricanes, sea level rise, and human activities—that have driven extensive land loss. Since the 1930s, approximately 4920 km^2^ of land in the MRD has disappeared, and projections estimate an additional 10,670 km^2^ may be lost over the next 50 years [30]. Within this landscape, *P. australis* dominates and forms vast monocultures [28,40]. There are multiple lineages of *P. australis* in the MRD with the Delta lineage (haplotype M1) being the most abundant in the MRD; therefore, we focused on the Delta lineage only [27,40]. However, in 2016 a major dieback of *P. australis* was reported across the MRD. Symptoms of dieback include retreat from deep-water habitats, reduced stem growth, premature leaf senescence, and the widespread occurrence of dead or decaying rhizomes [41,42,43,44]. This dieback poses serious ecological and economic risks: it accelerates land conversion to open water by reducing sediment capture and promotes the infilling of navigation channels critical for commerce and transportation [45]. In response, our multidisciplinary team has investigated the causes and consequences of the phenomenon. Findings suggest that dieback is driven by multiple interacting stressors, including saltwater intrusion from channel deepening, sea level rise, prolonged flooding, competition with other invasive plants and outbreaks of invasive pests, among others [28,30,31,46].

*Nipponaclerda biwakoensis* is a sap-sucking specialist of *P. australis* native to Japan, China, and Korea [47,48], it now reaches mid-summer abundances of ~150 individuals per meter of stem in the MRD [49]. Even relatively low densities (~20 per m of stem) have been shown to significantly reduce *P. australis* biomass production in common-garden studies [31,49]. Despite these well-documented impacts, it remains unclear how this invasive insect spreads so rapidly among stands. Movement of crawlers may occur both actively, through short-distance crawling, and passively via wind, water currents, or hitchhiking on other organisms. Support for passive, long-distance dispersal of scales comes from Lee et al. [29] who documented the spread across open water into restored *P. australis* patches located 20 m from the nearest infested stand in only 11 months. Understanding the mechanisms underlying crawler movement and their settling preferences is therefore critical for developing effective management strategies.

### 2.2. Scale and Parasitoid Density Patterns in MRD P. australis Stems

For this study, we focused on the *P. australis* Delta lineage (haplotype M1), derived from the Mediterranean region, closely related to European lineages, and found only in Louisiana [50]. Ten *P. australis* stems from the Delta lineage were randomly selected from each of 11 field populations, for a total of 110 stems, in Pass-a-Loutre channel of the Mississippi River Delta, Louisiana, USA in July 2023 (Table S1). Individual stems were cut at the water line and taken back to the lab at Louisiana State University.

The length of each of the ten stems per population source was measured (mean_Height_ = 2.17 m ± 0.015 SE; n = 110) and divided into three equal length sections measuring approximately 0.72 m. We designated bottom, middle, and top sections for stems collected from the field due to their substantial height. The number of gravid female and parasitized scales were counted per node. Specifically, gravid female scales are reproductively mature and carry fully developed scale eggs. Several hymenopteran parasitoids (five different species) attack gravid female scales in the MRD by ovipositing wasp eggs in scale eggs. Parasitoid wasps are responsible for 10–50% mortality of *N. biwakoensis* scales in Louisiana [34]. Parasitized scales are distinguished from gravid female scales by dark coloration and distinct compartments. Total number of parasitized scales were counted; however, we did not rear parasitoids for subsequent identification. Overall density was then calculated as the total number of *N. biwakoensis* scales per meter of stem for each stem section and for the entire *P. australis* stem length. Percent parasitized was calculated as the number of parasitized scales divided by the total number of scales.

### 2.3. Plant Nutritional and Defensive Traits of P. australis Stems

To assess factors influencing crawler settling, we examined whether nutritional and defensive trait levels vary between the top and bottom sections of plant stems. Source populations from the Delta lineage of *P. australis* in the MRD have been maintained at Louisiana State University since 2010 [51,52]. By standardizing growing conditions for all source populations, maternal effects that might influence plant growth and chemistry were minimized. Rhizomes used in all assays and experiments were collected from eight source populations (Table S2) and planted in peat-based garden soil (Cleggs Nursery, Baton Rouge, LA, USA) in 2.37 L plastic pots. The pots of all the same source population were placed in 1.2 m diameter plastic pools filled with tap water to a depth of ≈15 cm. One week after establishment, each pool was supplemented with 28 g of Osmocote (9-month, slow-release 15-9-12 N-P-K). To each pool, we also added a 36 mL solution comprising 45 g of Miracle Gro (24-8-16 NPK, The Scotts Miracle-Gro Co., Marysville, OH, USA), 132 mL of Liquinox (iron and zinc supplement; Liquinox Co., Orange, CA, USA) and 11.3 L of water. Individual plants were established for one month at the LSU AgCenter Plant Materials Center, Baton Rouge, Louisiana, USA before being used for chemical analyses and crawler-choice experiments. Plants grown in the greenhouse were maintained at 24–32 °C during the day and 18–24 °C at night, with supplemental lighting extending the photoperiod to 14 h.

From our established plants, ten replicates from each of the eight source populations were harvested and measured for % carbon, % nitrogen, and water content as proxies for nutritional traits. These plants were partitioned into bottom and top sections, as they grew to roughly 1 m to fit inside the cage, and dried in a drying oven at 21 °C. Stems were then ground and analyzed for percent carbon and nitrogen at the LSU AgCenter’s Soil Testing & Plant Analysis Lab in Baton Rouge, LA, USA. The lab quantified % C and % N by dry combustion using a LECO Carbon/Nitrogen Dumas Analyzer (LECO Corporation, St. Joseph, MI, USA). Water content was measured by subtracting the dry mass from the fresh mass of each section. Fresh mass was measured immediately and then the stems were dried in an oven at 62 °C. Dry mass was measured two weeks later.

We used another ten replicates from each of the eight source populations to quantify total phenolics as a proxy for defensive traits. Each stem was cut 1 cm from both the bottom and top ends. Those 1 cm cuttings were used for phenolic determinations and ranged from 250 to 500 mg in dry weight. Each cutting was extracted by placing fresh (undried) cuttings into a scintillation vial with 5 mL of a 1:1 methanol and water mixture for phenolic extraction. The vials were stored in the dark for three days before being used for estimates of total phenolics. Total phenolic content was determined using the Folin–Ciocalteu assay [53], with gallic acid as a standard. From each vial of phenolic extract, 0.10 mL of the liquid was placed in a test tube. Then, 2.65 mL of water was added to the test tube for a total volume of 2.75 mL. Each test tube then received 0.50 mL of the Folin–Ciocalteu reagent (diluted 1:1 with water) (Sigma-Aldrich, St. Louis, MO, USA). After five minutes, 0.50 mL of 20% Na_2_CO_3_ solution was added to each test tube and then vortexed. After 90 min, the solutions were transferred to cuvettes and absorbance at 720 nm was measured using a dual-beam spectrophotometer (VWR UV-6300PC, Radnor, PA, USA). Test tubes containing 2.75 mL of water with no phenolics were prepared in a similar manner and used as blanks in the spectrophotometer. A standard curve was prepared with caffeic acid as the standard using an identical procedure.

### 2.4. Scale Preference Assay: Potted Plant Choice Test

In the greenhouse, we conducted two types of choice assays to test whether scales preferred settling on the bottom or top stem sections. In March 2023, twenty-four rhizomes from each of three MRD sites were potted in a peat-based garden soil (Table S3, Cleggs Nursery, Baton Rouge, LA, USA) in 2.37 L plastic pots and established in a greenhouse at the LSU AgCenter Plant Materials Center near the campus of Louisiana State University in Baton Rouge, Louisiana. Plants grown in the greenhouse were maintained at 24–32 °C during the day and 18–24 °C at night, with supplemental lighting extending the photoperiod to 14 h. Plants were used for the experiment once the stem reached roughly 1 m high. Gravid female *N. biwakoensis* were collected from source populations across the MRD and immediately taken to the greenhouse to begin the inoculation portion of the experiment.

On 11 April 2023, twelve potted *P. australis* plants were placed individually in 61 × 61 × 91 cm mesh collapsable mesh cages using the same greenhouse conditions previously described (BioQuip, Compton, CA, USA). All stems in a pot, except for the longest one, were cut at the base and removed so that only a single stem remained in each pot. The total height of each stem was recorded. The total number of nodes and distance between nodes were also recorded. The node closest to the middle of the stem was marked with permanent marker to denote the dividing point between the upper and lower half of the stem, resulting in three sections—bottom, middle, and top. Each plant was then inoculated by carefully placing three gravid adult female *N. biwakoensis* scales at the middle node by inserting between the leaf sheath and stem. One month following inoculation, the final plant height (m), total number of nodes, and the number of new scales were quantified across the three stem sections. Gravid females were introduced at the middle section and allowed to disperse either upward or downward along the stem. The distance (cm) from the middle mark to each scale at a node was recorded, with downward movement scored as negative and upward movement scored as positive.

This same protocol was repeated on 21 April 2023, using the remaining 12 plants. However, out of 24 experimental plants infested over the two experiments, only nine plants showed successful settling of nymphs and yielded useful data. The low hatch rate of female scales is likely attributable to their fragility during transfer; scraping individuals from the stem often damages or detaches the penetrating mouthparts, which may be fatal. An additional possibility is that some females were moved before their eggs had fully matured. Overall scale densities were calculated as the total number of *N. biwakoensis* scales per meter of *P. australis* stem for each of the 9 stems. Then, individual densities were calculated for the bottom, middle, and top sections of the stems.

### 2.5. Scale Preference Assay: Petri Dish Choice Test

We conducted a second choice-assay to test whether crawlers prefer bottom or top sections of the vertical stem. We used paired sections obtained from the base of the plant and from the tip of the shoot (bottom and top cuttings, respectively) measuring 3.5 cm from individual *P. australis* stems and placed in a centrifuge tube filled with water. There were eight replicates from each of the same three source populations used in the potted plant choice-assay described above. Three gravid females were placed in the center of a Petri dish (150 mm Fisher Scientific, Waltham, MA, USA). Each pair of cuttings was randomly placed either to the left or right, at equal distances from the central cluster; Petri dishes were covered and left alone for a month. All Petri dishes were kept in the same environmental conditions (temperature = 18.3 °C, humidity = 70%, 12 h light/12 h dark). Paired bottom and top of the stem cuttings were assessed for scale establishment a month later. Out of 24 experimental plants infested over the two experiments, eleven plants showed successful settling of nymphs and yielded useful data. Female hatch rates were likely low because scraping them from stems can damage the embedded mouthparts, and some individuals may have been moved before eggs were fully developed. The stem section on which the majority proportion of scales (mean_Bottom_ = 18.6 scales per m of stem, mean_Top_ = 3.5 scales per m of stem) were found was recorded as the preferred section for each of the 11 dishes showing successful settling of nymphs.

### 2.6. Statistical Analysis

We used a generalized linear model (GLM) with stem section and population of source rhizome as the fixed effects for all analyses. We used a Poisson distribution for all scale and parasitized scale response variables (count data) because the data was over-dispersed and right skewed. All models were followed up with a likelihood ratio test and model residual deviance was lower than null model deviance [54]. We tested whether the different thirds of the stem were equally occupied by scales. For parasitized scales models, we included scale densities in addition to stem position as a factor in the model to test for density-dependence within stem sections. Similarly, a GLM was used for all models during trait quantification where the trait measurement itself was the response variable. For carbon, nitrogen, water content, and total phenolics, the fixed effects were stem position and source plant population. We also used a GLM for the potted plant choice assay. The response variable was number of scales per node, and the predictor variables were stem position (bottom, middle or top) and population of source plant. Finally, we used a binomial test for the Petri dish choice assay because our data were binary and we tested if the observed frequency of the crawlers choosing the bottom section of the stem significantly different from random (*p* = 0.05). Effects sizes were calculated for all models. Pairwise comparisons among treatment levels (i.e., different stem sections) were assessed using Tukey’s Honest Significant Difference post hoc test using the emmeans package in R (version 4.1.2) [55]. All of the analyses were conducted using R statistical programming [56].

## 3. Results

### 3.1. Scale and Parasitoid Density Patterns in MRD P. australis Stems

**Figure 1:**
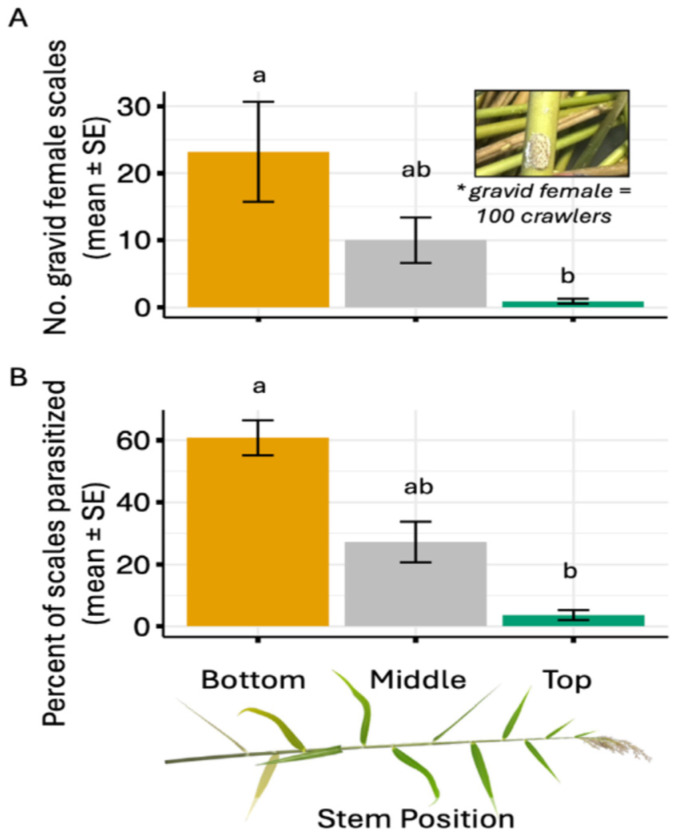
Mean (±SE) (**A**) number of gravid female scales and (**B**) percentage of parasitized scales present in the bottom, middle and top section of stems collected from the Mississippi River Delta, Louisiana during July 2023. Different letters above columns indicate significant differences among treatments based on Tukey’s HSD test (*p* < 0.05). The asterisk represents a note about gravid females.

Among field-collected stems, we found two times more scales on the bottom sections of stems compared to the middle sections, and 20 times more scales on the bottom than top sections of the stem (Figure 1A, *ANOVA Likelihood*: *χ^2^* = 6.43, df = 2, *p* = 0.040). Similarly, we found 12 times more parasitized scales on the bottom section of the stem compared to the middle and top sections (Figure 1B; *Tukey Comparison Test*: *estimate =* −17.23, *z-value* = −3.56, *p* = 0.001). However, we found parasitism was density dependent and influenced by overall scale abundance (mixed model: Estimate = 0.85, df = 23.80, *p* < 0.001).

### 3.2. Plant Nutritional and Defensive Traits of P. australis Stems

**Figure 2:**
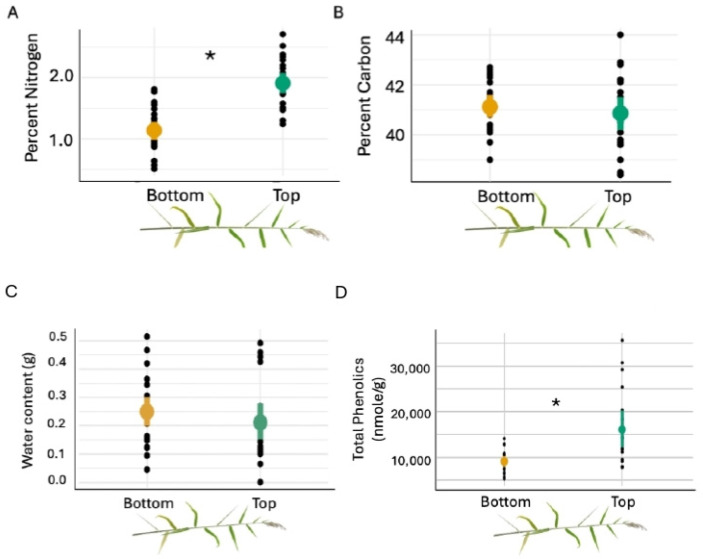
Mean (±SE) of plant nutritional and defensive traits (**A**) % nitrogen, (**B**) % carbon, (**C**) water content, and (**D**) total phenolics between the bottom and top sections along the stem of *Phragmites australis* (Delta lineage) grown under standardized greenhouse conditions. Plants were grown under standardized greenhouse conditions. Black points represent individual stem sections and asterisks represent significant difference (*p* < 0.05).

We quantified carbon, nitrogen, and water content along the stem length as a proxy for nutritional quality. We found that percent nitrogen was 50% higher in the top section of the stem compared to the bottom section (Figure 2A, *ANOVA Log Likelihood*: *χ*^2^ = 32.35, df = 1, *p* < 0.001). However, there was no significant difference in percent carbon (Figure 2B, *ANOVA Log Likelihood*: *χ*^2^ = 0.4326, df = 1, *p* = 0.51) or the amount of water (Figure 2C, *t*-test: t-value= −1.13, df = 36.86, *p* = 0.27) between the top and bottom sections of the stem length. For total phenolics, our proxy for plant defense, we found the top section had 47% more total phenolics compared to the bottom section of the vertical stem (Figure 2D, *ANOVA Log Likelihood*: *χ*^2^ =9.0294, df = 1, *p* = 0.0027).

### 3.3. Behavior Assays: Whole-Plant and Petri Dish Choice Tests

**Figure 3:**
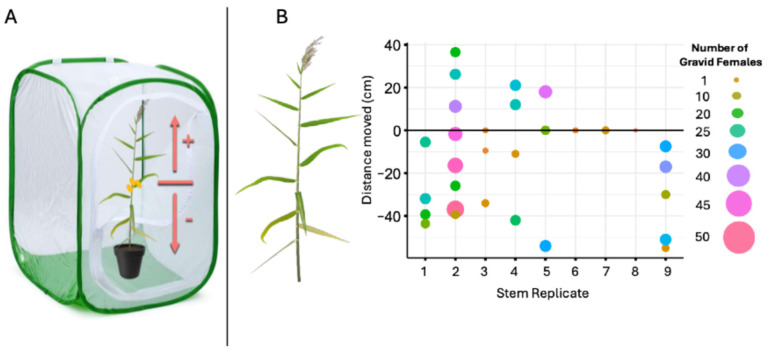
Crawler settling preferences using a whole-stem choice assay in an enclosed greenhouse experiment. (**A**) the experimental setup where individual caged *Phragmites australis* (Delta lineage) plants were inoculated with 3 gravid females (orange ovals) placed in the middle of the plant. Scale settlement was quantified following one month. (**B**) Scale density along the length of individual *P. australis* stems (n = 9). Density plot where each dot represents a cluster of gravid female scales around the nearest node. Size and color of dots represent number of scales within cluster. The *y*-axis is the distance to the farthest scale within the cluster around the nearest node.

**Figure 4:**
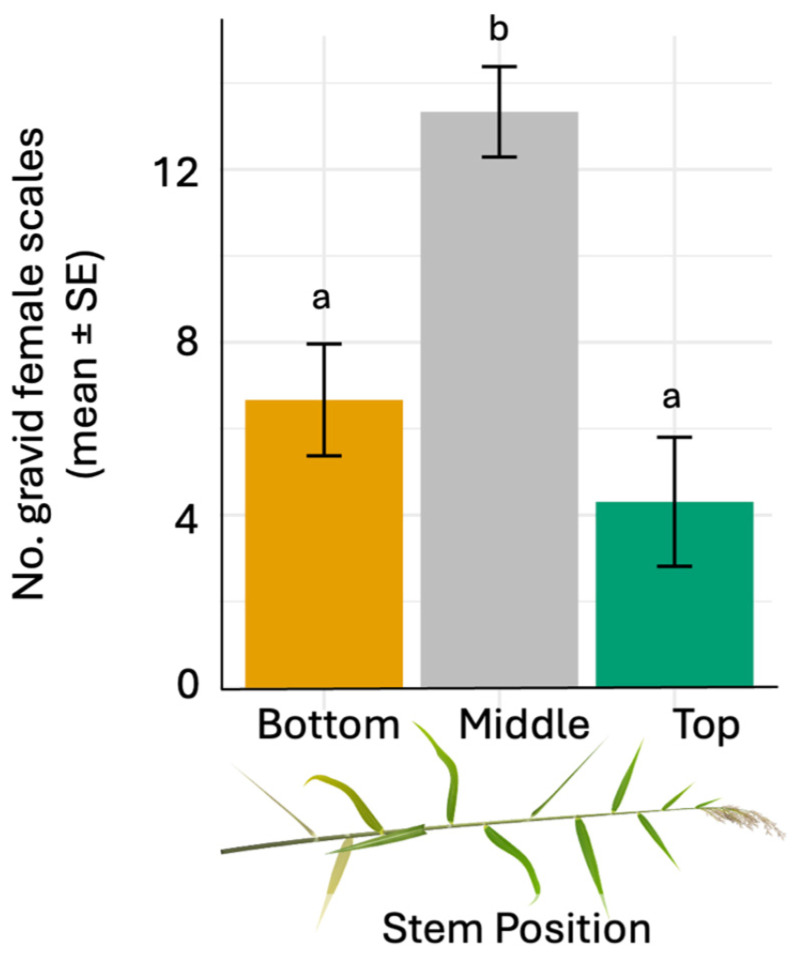
Average (±SE) number of gravid females located in the bottom, middle and top of *Phragmites australis* (Delta lineage) stems. Three gravid female scales were placed at the middle of a caged plant, and following one month of establishment, mature scale clusters were counted and averaged across sections of the vertical stem—bottom, middle, top. Different letters above columns indicate significant differences among treatments based on Tukey’s HSD test (*p* < 0.05).

**Figure 5:**
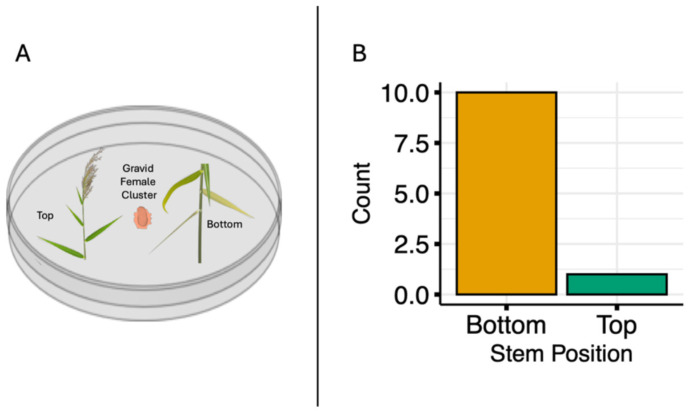
(**A**) Choice experiment setup in which 3 gravid female scales were placed in the center of a Petri dish with a bottom and top cutting from the same *Phragmites australis* (Delta lineage) stems. (**B**) The number of trials (out of 11 total) in which the majority of crawlers settled in the bottom versus top stem section.

Using a whole-plant choice assay (Figure 3A), we found two times more gravid scales in the middle section of the stem compared to either the bottom or top sections of the stem (Figure 3B and Figure 4, *ANOVA likelihood test*: *χ*^2^ = 25.387, df = 2, *p* <0.001). There was no difference in the number of scale clusters located on the bottom compared to the top of the stem. Using a Petri dish choice assay, we found that, in 10 out of 11 replicates, the majority of crawlers chose the bottom over the top section; a significant difference (Figure 5, Binomial Test: *p* value = 0.012; 95% CI [0.0023, 0.41]). Thus, the final count depicts the section (bottom or top) where the majority of crawlers settled (mean_Bottom_ = 18.6 scales per m of stem, mean_Top_ = 3.5 scales per m of stem).

## 4. Discussion

*Phragmites australis*, a key stabilizer of the Mississippi River Delta, has suffered major dieback following invasion by the scale insect *N. biwakoensis*. Despite its rapid Gulf Coast spread since 2016 [34], little is known about how this insect disperses within individual plants. We tested the movement of crawlers (the mobile life stage) to determine their settling preferences along *P. australis* stems. Our results revealed settlement patterns favoring the middle and lower stem sections in both lab and field assays, higher parasitism rates at the stem base, and lower nitrogen and phenolic concentrations in these tissues. Together, these findings suggest that settlement patterns emerge from an interplay of habitat structure, nutritional quality, and the presence of parasitoids, factors we consider in turn below.

Patterns of *Nipponaclerda biwakoensis* settlement along the basal stems of *P. australis* likely emerge from an interplay between habitat structure, host density, and natural enemy dynamics. Elevated parasitism near the stem base coincides with higher scale densities, suggesting that parasitoids may be responding to, rather than driving, crawler settlement. In many systems, parasitism tends to increase where host densities are highest, reflecting density-dependent parasitism rather than enemy-driven settlement [57,58]. In a previous study of *N. biwakoensis* in its native range, Kaneko [13] observed that *N. biwakoensis* and its parasitoids both concentrate at the base of *P. australis* stem. Similarly, *Pseudococcus maritimus* and related mealybugs aggregate in the basal portions of grapevines and tree trunks where humidity and structural refuge improve survival, even under higher parasitism pressure [59,60]. Collectively, these studies suggest there is a positive density-dependent response by the parasitoids.

Importantly, the parasitoid community is composed of at least five species, including both specialists and generalists, each exploiting *N. biwakoensis* at a different life stage [34]. This community is also taxonomically mixed: several parasitoid species were already present in the Mississippi River Delta prior to the scale’s arrival, whereas at least one species appears to have been inadvertently introduced alongside *N. biwakoensis*. These differences in host specificity, origin, and life-history strategy likely generate heterogeneous selective pressures across the stem profile, complicating simple assumptions about enemy-driven settlement. Because we did not rear parasitoids to confirm species identity, our inferences necessarily treat “parasitoid pressure” as a composite effect. This limitation underscores the need for species-level identification and life-history characterization to disentangle how individual parasitoid taxa may differentially influence crawler distribution and survival. At the same time, other mechanisms unrelated to natural enemies—such as desiccation avoidance, microclimatic buffering, structural refuge within dense basal stems, or olfactory interference from swampy, anaerobic soils—may also shape settlement behavior.

While the observed overlap between parasitoid activity and settlement at the stem base is intriguing, there is currently no direct evidence that natural enemy pressure causally influences crawler choice. In fact, if parasitoids impose stronger selection near the base, one might expect adaptive shifts favoring settlement higher up the stem, where exposure may be greater but enemy pressure lower. To resolve this uncertainty, future experiments could manipulate parasitoid presence, alter stem density, or modify microclimatic and olfactory cues to test whether settlement preferences change when enemy access is restricted. Such experiments would clarify whether natural enemies actively shape within-plant distribution patterns or simply exploit host-dense regions. Ultimately, disentangling these biotic and structural drivers is key to understanding how *N. biwakoensis* populations establish, persist, and spread within *P. australis* stands.

Given that *N. biwakoensis* settling preference could not be simply attributed to gravitropic behaviors of the crawlers, nutritional and defensive traits appear to play a key role in where *N. biwakoensis* crawlers settle. Scale densities were consistently higher on stem sections with lower nitrogen and reduced total phenolic concentrations. In the choice assay, when given equal-sized cuttings from the top and bottom of the stem at the same level, settlement was significantly greater on the lower portions—tissues that were less nutrient-rich and less chemically defended. This pattern aligns with patterns observed in other sedentary insect herbivores, where herbivores favor sites with weaker chemical defenses or more favorable microclimates over nutrient-rich tissues [61]. Such patterns are consistent with the Growth–Defense Tradeoff Hypothesis, which posits that rapidly growing, nutrient-rich tissues invest more heavily in chemical defenses to deter herbivory [16,24,25]. In *P. australis*, elevated phenolic defenses in newer, nitrogen-rich growth may outweigh its nutritional advantages, leading crawlers to settle on older, less-defended tissues. Similar to findings in other concealed or sessile herbivores, dense basal stems likely provide both physical refuge and microclimatic stability, reducing exposure to predators and desiccation [62,63]. Collectively, these results suggest that structural and environmental concealment may enhance survival, while reduced chemical defenses increase feeding and establishment opportunities, together shaping the within-plant distribution of *N. biwakoensis*.

Understanding the density-dependent aggregation of *N. biwakoensis* toward the base of *P. australis* stems provides key insight into how this insect spreads—moving vertically within stems and horizontally among shoots—to transform localized scale colonies into extensive plant die-offs across marsh landscapes. Our results revealed that *N. biwakoensis* settlement was concentrated in the middle and lower stem sections, where nitrogen and phenolic concentrations were reduced and parasitism rates were highest. These patterns suggest that early crawler settlement is biased toward microhabitats that optimize survival and feeding conditions [62,63]. Increasing local density, higher parasitism, and declining resource quality in basal refuges likely trigger upward crawler dispersal to less crowded tissues, consistent with density-dependent movement observed in other scale systems [59,64]. As crawlers move higher along stems, they may become more exposed to wind currents, potentially facilitating dispersal to new shoots or patches as basal resources are depleted. At the same time, the basal zone may also promote horizontal spread across neighboring stems. In dense *P. australis* stands, physical contact among basal shoots, overlapping leaf sheaths, and hydrological connectivity via surface water create pathways for lateral crawler transfer. Crawlers emerging near the stem base are well positioned to move horizontally by crawling, wind, or water currents, facilitating colonization of adjacent stems and clumps. Similar cross-stem dispersal has been documented in other sessile insects where crowding and habitat continuity accelerate local infestation growth [59]. Thus, the combination of favorable microhabitat conditions, nutrient–defense gradients, and structural connectivity likely drives both vertical and horizontal expansion of *N. biwakoensis* colonies within *P. australis* stands. These mechanisms together explain how initially localized infestations can coalesce into large, persistent patches characteristic of *P. australis* die-off regions across the Mississippi River Delta.

## Data Availability

The data that support the findings of this study are openly available in [FigShare] at [https://doi.org/10.6084/m9.figshare.30495368].

## Supplementary Materials

The following supporting information can be downloaded at: https://www.mdpi.com/article/10.3390/insects17010009/s1.
