# Supplementary material for: Plant Chemistry and Enemy Pressure Shape Within-Stem Distribution of the Invasive Scale Nipponaclerda biwakoensis
Source: Insects. 2025 Dec 20;17(1):9. doi: 10.3390/insects17010009 (PMC12842001; doi:10.3390/insects17010009)
Supplement: Supplementary file 1 [file insects-17-00009-s001.zip › insects-3988779-supplementary.pdf]

Supplement

Plant chemistry and enemy pressure shape within-stem distribution of the invasive scale *Nipponaclerda biwakoensis*

Andrea E. Glassmire*, James T. Cronin, Rodrigo Diaz, Alexis Desoto, Emily Shapiro, Alex Gaffke, Joshua S. Snook, and Michael Stout

*Corresponding author: glassmi5@msu.edu

Table of Contents

Table S1. Populations of *P. australis* (Delta lineage) used to assess scale and parasitism along stems collected from the Mississippi River Delta

Table S2 – Populations of *P. australis* (Delta lineage) used in the nutritional and defensive plant trait analysis

Table S3 – Populations of *P. australis* (Delta lineage) used for both whole-plant and Petri dish scale choice-assay

Table S4 – Summary statistics for gravid female scales densities as a function of bottom, middle, and top sections from MRD collected stems

Table S5. Summary statistics for percent of scales parasitized as a function of bottom, middle, and top sections from MRD collected stems

Table S6. Summary statistics for water content as a function of bottom and top sections

Table S7. Summary statistics for % nitrogen as a function of bottom and top sections

Table S8. Summary statistics for % carbon as a function of bottom and top sections

Table S9. Summary statistics for total phenolics as a function of bottom and top sections

Table S10. Summary statistics for scale whole-plant choice assay as a function of bottom, middle, and top sections

Table S11. Summary statistics for scale petri dish choice assay as a function of bottom and top sections of individual *P. australis*.

Table S1. Populations of *P. australis* (Delta lineage) used to assess scale and parasitism along stems collected from the Mississippi River Delta

**Population**	**Latitude**	**Longitude**	**Date of sampling**
DEH	29.126	89.210	7/18/22
ECM	29.194	-89.296	7/20/22
PLM	29.130	-89.230	8/1/22
EARL1	29.194	-89.296	6/1/23
20.897469, -90.262489	20.897	-90.262	6/1/23
29.140113, -89.189775	29.140	-89.190	7/13/23
29.206866, -89.230090	29.207	-89.230	7/13/23
AN7	29.038	-89.219	7/27/23
BD01_DD	29.139	-89.220	8/23/23
BD07_DH dredge	29.105	-89.207	8/23/23
JC2	29.164	-89.167	8/23/23

Table S2. Populations of *P. australis* (Delta lineage) used in the nutritional and defensive plant trait analysis. Individual plants were grown in the greenhouse. The bottom and top sections of individual stems were used for carbon, nitrogen, water content, and total phenolics analyses.

**Population**	**Latitude**	**Longitude**
BD07-DH dread	29.105	-89.207
ECM	29.194	-89.296
PLM	29.130	-89.230
EARL1	29.194	-89.296
20.897469, -90.262489	20.897	-90.262
29.140113, -89.189775	29.140	-89.190
29.206866, -89.230090	29.207	-89.230
DEH	29.126	89.210

Table S3. Populations of *P. australis* (Delta lineage) used for both whole-plant and Petri dish scale choice-assay. Individual plants were established in the greenhouse.

Population	Latitude	Longitude
BD07-DH dredge	29.105	-89.207
ECM	29.194	-89.296
EARL1	29.194	-89.296

Table S4. Summary statistics for gravid female scales densities as a function of bottom, middle, and top sections of individual *P. australis*

Fixed Effects	Estimate	Std. Error	df	*t value*	*P value*
Intercept	7.475	1.778	27	4.204	0.00026 ***
Middle Section	-4.885	2.515	27	-1.943	0.063
Top Section	-6.01	2.515	27	-2.39	0.024 *

Table S5. Summary statistics for percent of scales parasitized as a function of bottom, middle, and top sections of individual *P. australis*.

Fixed Effects	Estimate	Std. Error	df	*t value*	*P value*
Intercept	17.17	3.97	26.945	4.325	0.00019 ***
Center Section	-6.532	5.524	18	-1.182	0.25
Top Section	-15.412	5.524	18	-2.79	0.012 *

Table S6. Summary statistics for water content as a function of bottom, middle, and top sections of individual *P. australis*.

Fixed Effects	Estimate	Std. Error	df	*t value*	*P value*
Intercept	0.48	0.036	4.26	13.28	0.00013 ***
Top Section	-0.051	0.045	36.86	-1.13	0.26

Table S7. Summary statistics for % nitrogen as a function of bottom, middle, and top sections of individual *P. australis*.

Fixed Effects	Estimate	Std. Error	df	*t value*	*P value*
Intercept	0.011	0.0012	3.42	9.59	0.0014 **
Top Section	0.0077	0.0011	40.097	6.92	2.41e-08 ***

Table S8. Summary statistics for % carbon as a function of bottom, middle, and top sections of individual *P. australis*.

Fixed Effects	Estimate	Std. Error	df	*t value*	*P value*
Intercept	0.41	0.0038	3.82	106.99	8.9e-08 ***
Top Section	-0.0025	0.0039	40.12	-0.65	0.52

Table S9. Summary statistics for total phenolics as a function of bottom, middle, and top sections of individual *P. australis*.

Fixed Effects	Estimate	Std. Error	df	*t value*	*P value*
Intercept	9237.88	2222.85	2.95	4.156	0.026 *
Top Section	6961.16	2171.84	29.72	3.205	0.0032 **

Table S10. Summary statistics for scale whole-plant choice assay as a function of bottom, middle, and top sections of individual *P. australis*.

**Fixed Effects**	**Estimate**	**Std. Error**	**df**	***t value***	***P value***
Intercept	0.99	0.28	10.19	3.49	0.0057 **
Middle Section	0.68	0.18	91.31	3.68	0.00039 ***
Top Section	-0.26	0.17	91.38	-1.58	0.12

Table S11. Summary statistics for scale petri dish choice assay as a function of bottom and top sections of individual *P. australis*.

**Estimate**	**95% Confidence Interval**	**Sample Size**	***P value***
0.0909	[0.0023, 0.41]	11	0.012
